# Antibacterial Activity of Zinc Oxide Nanoparticles Loaded with Essential Oils

**DOI:** 10.3390/pharmaceutics15102470

**Published:** 2023-10-15

**Authors:** Ludmila Motelica, Bogdan-Stefan Vasile, Anton Ficai, Adrian-Vasile Surdu, Denisa Ficai, Ovidiu-Cristian Oprea, Ecaterina Andronescu, Gabriel Mustățea, Elena Loredana Ungureanu, Alina Alexandra Dobre

**Affiliations:** 1 National Research Center for Micro and Nanomaterials, National University of Science and Technology POLITEHNICA Bucharest, Splaiul Independentei 313, 060042 Bucharest, Romania; motelica_ludmila@yahoo.com (L.M.); ecaterina.andronescu@upb.ro (E.A.); 2 National Research Center for Food Safety, National University of Science and Technology POLITEHNICA Bucharest, Splaiul Independentei 313, 060042 Bucharest, Romania; 3 Faculty of Chemical Engineering and Biotechnologies, National University of Science and Technology POLITEHNICA Bucharest, 1-7 Polizu St., 011061 Bucharest, Romania; 4 Academy of Romanian Scientists, Ilfov Street 3, 050044 Bucharest, Romania; 5 National R&D Institute for Food Bioresources—IBA Bucharest, Dinu Vintila Street 6, 021102 Bucharest, Romania

**Keywords:** ZnO, citronella, orange, thyme, lavender, grapefruit, bergamot, cinnamon, rosemary, minzol, limette, antimicrobial

## Abstract

One major problem with the overuse of antibiotics is that the microorganisms acquire resistance; thus the dose must be increased unsustainably. To overcome this problem, researchers from around the world are actively investigating new types of antimicrobials. Zinc oxide (ZnO) nanoparticles (NPs) have been proven to exhibit strong antimicrobial effects; moreover, the Food and Drugs Administration (FDA) considers ZnO as GRAS (generally recognized as safe). Many essential oils have antimicrobial activity and their components do not generate resistance over time. One of the drawbacks is the high volatility of some components, which diminishes the antimicrobial action as they are eliminated. The combination of ZnO NPs and essential oils can synergistically produce a stronger antimicrobial effect, and some of the volatile compounds can be retained on the nanoparticles’ surface, ensuring a better-lasting antimicrobial effect. The samples were characterized with X-ray diffraction (XRD), transmission electron microscopy (TEM), scanning electron microscopy (SEM), Fourier transform infrared spectroscopy (FTIR), ultraviolet-visible spectroscopy (UV-Vis), and thermal analysis (TG-DSC) coupled with analysis of evolved gases using FTIR. The ZnO NPs, with a size of ~35 nm, exhibited a loading between 1.44% and 15.62%—the lower values were specific for limonene-containing oils (e.g., orange, grapefruit, bergamot, or limette), while high values were obtained from cinnamon, minzol, thyme, citronella, and lavender oils—highlighting differences among non-polar terpenes and alcohol or aldehyde derivatives. The antibacterial assay indicated the existence of a synergic action among components and a high dependency on the percentage of loaded oil. Loaded nanoparticles offer immense potential for the development of materials with specific applications, such as wound dressings or food packaging. These nanoparticles can be utilized in scenarios where burst delivery is desired or when prolonged antibacterial activity is sought.

## 1. Introduction

One major problem with the overuse of antibiotics is that the microorganisms are acquiring resistance; thus the dose must be increased unsustainably [1]. To overcome this problem, new types of antimicrobials are being researched worldwide, from innovative synthetic organic compounds [2] to metallic and oxide nanoparticles [3], natural antimicrobials like plant extracts or essential oils [4], and synthetic peptides that can be designed to have antibacterial properties similar to antibiotics [5].

Zinc oxide (ZnO) nanoparticles (NPs) have been proven to exhibit strong antimicrobial effects, but at the same time U.S. Food and Drugs Administration (FDA) considers ZnO as GRAS (generally recognized as safe) [6]. The scientific literature is rich with numerous reports highlighting the antibacterial activity of ZnO nanoparticles against model pathogens, including both Gram-negative and Gram-positive strains. These nanoparticles have garnered significant interest due to their remarkable ability to inhibit the growth and proliferation of bacteria [7,8,9]. The antifungal activity is researched and reported much less, and usually in food-related applications. At the same time, the debate about the exact mechanism involved in the antimicrobial activity is still ongoing. The ZnO with its strong photocatalytic activity is expected to generate considerable levels of reactive oxygen species (ROS) under light irradiation [10], which are the main toxic species for microorganisms. However, ZnO NPs also exhibit antimicrobial activity under dark conditions, which supports alternative killing pathways. The most-often-mentioned ones are the mechanical damage to the cellular membrane and the internalization of NPs followed by the release of Zn^2+^ ions. All these factors promote ZnO NPs as an alternative microbicide agent in various domains, like food packaging, topical ointments, water treatment, and others [11,12,13].

Natural plant extracts and essential oils are also a major research topic, due to their multiple health benefits, which include antioxidant, antimutagenic, antiproliferative, or antimicrobial activities [14,15]; some of them—like citronella (lemongrass) essential oil—are classified as non-toxic biopesticides in the USA [16] while having a strong antifungal activity [17]. The antimicrobial activity of each essential oil derives from one or two main components. Additionally, minor components can also exhibit useful activities, and by synergism, the essential oil is usually more potent than the main components. The orange essential oil is composed mainly of D-limonene which is listed as GRAS by the FDA, but it is also considered a biopesticide and bug-repellant [18,19]. The antiseptic properties of thyme essential oil are generated by the main component, thymol [20]. The thyme essential oil was used as an antiparasitic or to treat minor wounds from ancient times due to its strong antimicrobial properties [21,22]. Lavender [23,24], grapefruit [25,26], bergamot [27,28], cinnamon [29,30], and rosemary [31,32] essential oils have strong antimicrobial activity and therefore are good candidates for innovative antibacterial and antifungal therapies and materials. Nevertheless, the essential oils must be loaded or encapsulated in a matrix, be it organic or inorganic, to ensure a longer release profile, and a sustained antimicrobial activity [33,34].

The nanoparticles can be loaded with various antimicrobials by multiple techniques—some being solvent-free and others involving a solvent. The solvent-free methods can be physical mixing, co-milling, melt method, or microwave irradiation. Moreover, solvent-mediated loading can be carried out using adsorption, incipient wetness impregnation, solvent evaporation, supercritical and liquid CO_2_ technology, one-pot drug loading and synthesis, chaperone assistance, etc. [35,36,37,38,39,40].

By combining the ZnO NPs with essential oils, the synergic activities result in either a stronger antimicrobial or a decrease in the amounts needed to combat microorganisms. Such loaded nanoparticles can be further used in topical ointments or other antimicrobial applications like food packaging [9]. By using essential oils loaded on ZnO NPs, some physical characteristics can also be improved, including retaining the volatile components into the mixture for a longer time, and slow release of compounds (due to interactions with nanoparticles surface) that can improve the availability of the essential oil components over time, providing a long-lasting antimicrobial activity.

In this study, we obtained polyhedral shape ZnO NPs with a diameter of ~35 nm, which were loaded with a series of the following essential oils: citronella, orange, thyme, lavender, grapefruit, bergamot, cinnamon, rosemary, minzol, and limette. Since these essential oils are provided as liquids, we chose a solvent-mediated loading mixed procedure, namely impregnation, followed by excess solvent evaporation. The ZnO NPs loaded with essential oils were characterized with thermogravimetry-differential scanning calorimetry (TG-DSC), Fourier transform infrared spectroscopy (FTIR), transmission electron microscopy (TEM), scanning electron microscopy (SEM), powder X-ray diffraction (XRD), ultraviolet-visible spectroscopy (UV-Vis) and fluorescence spectroscopy (PL).

The essential oil-loaded ZnO NPs were tested against a series of model pathogen bacterial strains—Gram-negative *Salmonella typhimurium* (ATCC 14028) and *Escherichia coli* (ATCC 25922), and Gram-positive *Staphylococcus aureus* (ATCC 25923) and *Listeria monocytogenes* (ATCC 19114)—which are one of the common causes of food poisoning [41,42,43,44,45]. The presence of both ZnO NPs and essential oils leads to an increase in antibacterial activity, indicating a synergic action.

## 2. Materials and Methods

Zinc acetate dihydrate (Zn(CH_3_COO)_2_·2H_2_O) with 99.9% purity was purchased from Merck (Merck Group, Darmstadt, Germany). The 1-butanol was obtained from Sigma (Redox Lab Supplies Com SRL, Bucharest, Romania). The nutrient growth medium used was TSA (Tryptone Soya Agar) type, produced by Oxoid Ltd. (Cheshire, UK).

Essential oils of citronella, orange, thyme, lavender, grapefruit, bergamot, cinnamon, rosemary, minzol, and limette were obtained from Carl-Roth (Karlsruhe, Germany), with major components being listed in Section 3.3.2.

All substances were used without further purification.

ZnO synthesis was performed as presented in [10]. Briefly, 5 g of zinc acetate dihydrate was poured in 50 mL of 1-butanol. The solution was refluxed for 24 h under continuous stirring. The flask was allowed to rest for 24 h at 25 °C. The precipitate was separated by decantation and subjected to repeated centrifugation. Between centrifugation, the powder was washed with absolute ethanol—the process being repeated thrice. Finally, the obtained white powder was dried in an electrical oven at 80 °C. An amount of 0.1 g of the obtained ZnO nanopowder was loaded with each essential oil (30 µL essential oil mixed with 100 µL ethanol). The mixture was ultrasonicated for 10 min and then evaporated at 40 °C in an electrical oven. The ZnO NPs loaded with essential oils were further used for microbiological assay. The sample labels are presented in Table 1.

Information about the crystallinity of the obtained ZnO nanopowder was obtained with a PANalytical Empyrean equipment (from Malvern PANalytical, Bruno, The Netherlands) using the equation λ_CuKα_ = 1.54184 Å. The X-ray diffractograms (XRDs) were recorded in the 2θ range 10–70°, with a step of 0.02° and a time per step of 0.1 s.

A Tecnai G2F30 S-TWIN high-resolution transmission electron microscope (TEM) from FEI (FEI Company, Eindhoven, The Netherlands) was used to record the micrographs of the ZnO NPs.

The surface morphology and microstructure of ZnO nanopowder were investigated with a scanning electron microscope, QUANTA INSPECT F50 (FEI Company, Eindhoven, The Netherlands).

Ultraviolet-visible (UV-Vis) reflectance spectrum was recorded with a V560 spectrophotometer from JASCO (Easton, PA, USA), using a 60 mm integrating sphere (ISV-469), between 200 and 900 nm.

An LS55 fluorimeter from Perkin Elmer (Perkin Elmer, Waltham, MA, USA), was used to record the fluorescence spectrum. The 320 nm radiation from the Xe lamp was used as an excitation wavelength. The emission spectrum was recorded between 350 and 700 nm, employing a 350 nm cut-off filter, with a scan speed of 200 nm min^−1^.

Thermogravimetry–differential scanning calorimetry was performed with a STA 449C F3 equipment, from Netzsch (Selb, Germany) coupled with a Tensor 27 FTIR from Bruker (Bruker Optik GmbH, Ettlingen, Germany), equipped with an internal thermostatic gas cell. A typical sample, weighing ~10 mg, was placed in an alumina crucible and was heated up at a rate of 10 °C∙min^−1^, up to 900 °C, in air.

A Nicolet iS50R spectrometer (Thermo Fisher Scientific, Waltham, MA, USA) device was used to record the Fourier transform infrared (FTIR) spectra, in attenuated total reflection (ATR). For every spectrum, an average of 32 scans was performed, from 400 to 4000 cm^−1^_,_ with a resolution of 4 cm^−1^.

To study in-vitro antibacterial activity, pure cultures of four reference bacterial strains obtained from the American Type Culture Collection (ATCC, Manassas, VA, USA) were used. These pathogenic bacteria (Table 2) naturally contaminate food products obtained under improper manufacturing conditions, producing food poisoning and infections. The nutrient growth medium used was TSA (Tripton Soy Agar), produced by Oxoid (Cheshire, UK).

Quantification of the working inoculum was carried out using the Plate Count technique, which involved the enumeration of the cells that determine the formation of colonies by cultivation on the nutrient culture medium. The qualitative screening of the sensitivity of different pathogenic bacteria to the samples of interest was performed with disk diffusion techniques on agar, adapted according to the standard issued by the Clinical and Laboratory Standards Institute (Wayne, PA, USA). In order to determine the antimicrobial activity of ZnO NPs, an adapted diffusion assay that follows the CLSI 2020 guidelines was employed. All antimicrobial experiments were performed in triplicates.

## 3. Results and Discussion

The obtained white ZnO nanopowder was investigated to determine the purity, composition, and morphology of the nanoparticles.

### 3.1. X-ray Diffraction Analysis (XRD)

**Figure 1:**
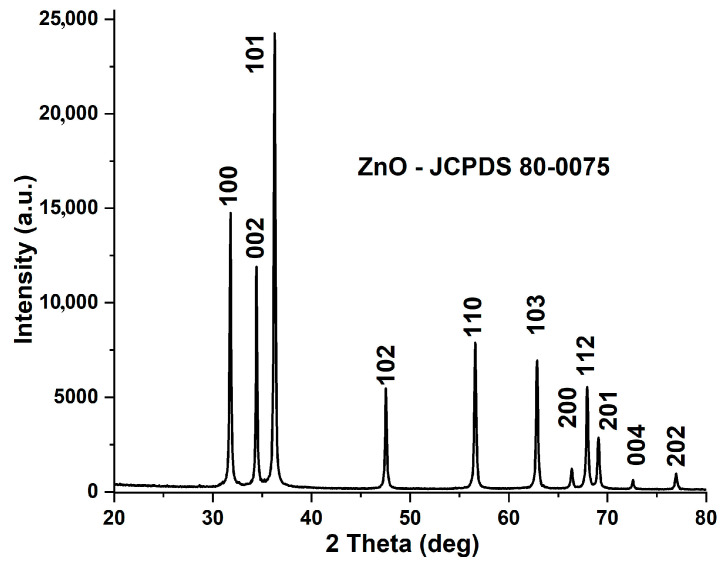
XRD pattern for the obtained ZnO nanopowder (JCPDS 80-0075).

The XRD analysis has revealed that the crystalline phase composed of only ZnO, Figure 1.

The XRD peaks were found at 2θ values (with Miller indices inside brackets): 31.78° (100), 34.43° (002), 36.25° (101), 47.56° (102), 56.61° (110), 62.85° (103), 66.39° (200), 67.95° (112), 69.09° (201), 72.60° (004), and 76.97° (202) [46]. The pattern was indexed as single-phase hexagonal wurtzite (ZnO) corresponding to JCPDS card no. 80-0075. Average crystallite diameter (D), microstrain (ε), and lattice parameters were calculated using Rietveld refinement, and the values are presented in Table 3.

The number of surface defects of the nanoparticles can be calculated as the dislocation density (δ) and is given by the length of dislocation lines in a volume unit. The δ value is calculated as 1/D^2^ (where D is the average crystallite diameter) [47]. The active centers, in the photocatalytic and antimicrobial activities, are represented by the defects on the nanoparticle surface. Such defects act as reactive oxygen species (ROS) generators; therefore, a sample with a high δ value can exhibit strong antimicrobial activity.

### 3.2. Scanning and Transmission Electron Microscopy

#### 3.2.1. Scanning Electron Microscopy (SEM)

**Figure 2:**
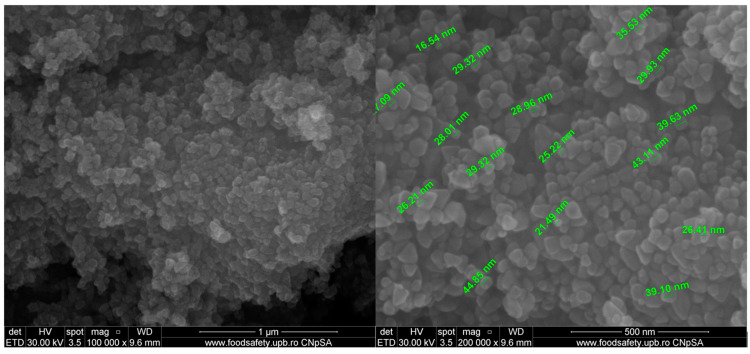
The SEM micrographs for the obtained ZnO nanopowder.

Morphology of the ZnO NPs was investigated with scanning electron microscopy (SEM). The SEM micrographs (Figure 2) indicate a uniform morphology, mostly polyhedral or triangular, with particle size averaging around ~30 nm, and a tendency to form soft agglomerates. The literature indicates that in the case of ZnO, smaller particles exhibit a higher antimicrobial activity [48]. Additionally, some microorganisms are more sensitive to the shape of nanoparticles, which can cause mechanical damage to the membrane and can lead to easier internalization [10].

#### 3.2.2. Transmission Electron Microscopy (TEM)

**Figure 3:**
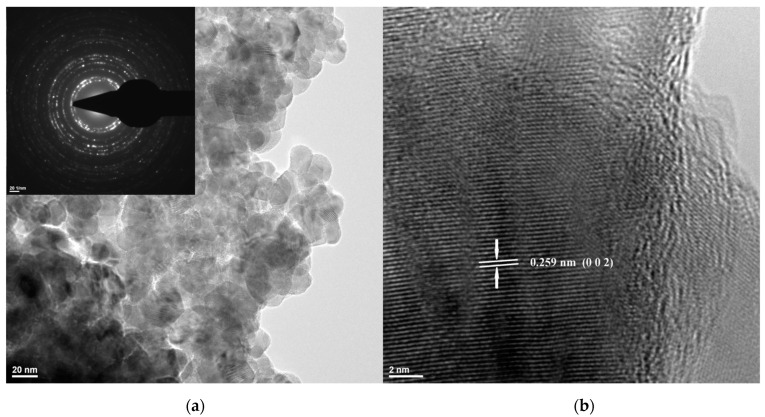
The TEM, SAED (inset) (**a**) and HRTEM (**b**) micrographs for the obtained ZnO nanopowder.

For a better image of ZnO NP size, shape, and morphology, TEM bright-field images were obtained (Figure 3), while the crystallinity of the sample was assessed with selected area electron diffraction (SAED). Images confirm that most ZnO NPs were polyhedral-shaped, with a diameter in the interval of 20–40 nm.

The SAED pattern obtained on the ZnO sample confirms that the only identified crystalline phase was the wurtzite form of ZnO. The high-resolution transmission electron microscopy (HRTEM) image (Figure 3) allows clear identification of atomic planes corresponding to the (0 0 2) Miller indices of wurtzite. Moreover, the regular succession of the lattice fringes, at distances of d = 2.59 Å, indicates the uniform crystalline structure, without the presence of an amorphous phase.

### 3.3. Spectroscopic Studies

#### 3.3.1. UV-Vis and Fluorescence (PL) Spectroscopy

**Figure 4:**
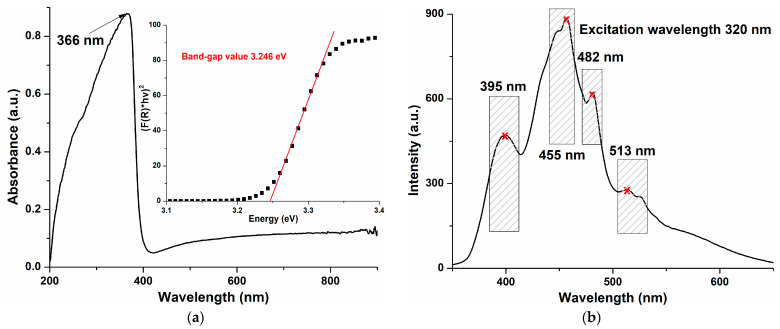
The UV-Vis spectrum and Tauc plot (inset) (**a**), and fluorescence spectrum (**b**) for the obtained ZnO nanopowder.

The UV-Vis spectrum for the ZnO nanopowders is presented in Figure 4a. The large and intense absorption band centered at 366 nm is typical for ZnO and similar to other literature reports [49]. Protected textiles or sunscreen cosmetics contain ZnO due to this strong absorption band in the UV domain [11].

ZnO is a direct band-gap semiconductor, with a wide band-gap of ~3.37 eV between the conduction and the valence bands. Therefore, a relatively large absorption band located in the ultraviolet domain is typical for the UV-Vis spectrum. The electron jump from the valence band to the conduction band generates this absorption peak, and its numerical value can be used to calculate the band-gap value of ZnO [50]. Some literature reports wrongly assign this band to a surface plasmon resonance, which does not exist in semiconductors like ZnO [51].

The band-gap value was determined using a Tauc plot (inset of Figure 4a) by applying the Kubelka–Munk function F(R) = (1 − R)^2^/2R—where R is the sample diffuse reflectance—and graphical extrapolation to [F(R)∙hν]^2^ = 0 [52]. The obtained value, 3.246 eV, was smaller than the theoretical value, indicating that the presence of additional electronic levels inside the band gap was most probably induced by the existence of various crystal defects [53]. Such defects were also responsible for the visible emission bands from the ZnO fluorescence spectrum (Figure 4b), called deep-level emission (DLE). The electronic levels induced inside the band-gap—by the defects like zinc vacancies (V_Zn_), oxygen anti-sites (O_Zn_), oxygen vacancies (V_O_), zinc interstitials (Zn_i_), or oxygen interstitials (O_i_)—generated the emission bands from 455, 482 and 513 nm, as previously reported in the literature [52,54,55].

On the contrary, the UV emission band, located near-band-edge (NBE) at 395 nm, was assigned to the recombination of free excitons. The recombination of excited electrons and holes could be blocked by the presence of defects, that could trap the free electrons. Therefore, a high density of surface defects can cause a decreased intensity of the NBE emission [56]. The production of ROS was increased by the surface defects, especially by the oxygen vacancies, and therefore their density was correlated with a higher antimicrobial activity under light irradiation [57].

#### 3.3.2. Fourier Transform Infrared (FTIR) Spectroscopy

The FTIR spectrum displayed two characteristic zones for the ZnO NPs. The very strong absorption band around ~420–450 cm^−1^ was assigned to the stretching vibrations of the Zn–O bond [8,58], while the weak peaks from ~610/680 cm^−1^ were assigned to Zn–OH bending vibrations [58,59] (Figure S1a). As previously reported in the literature, the band in the domain 400–500 cm^−1^ becomes very strong for the single phases of wurtzite, surpassing all other vibrations. Furthermore, the peaks corresponding to the bending of Zn–OH bonds remain at low intensity in the interval of 600–700 cm^−1^ [58]. Additionally, the peaks between 800 and 1100 cm^−1^ could be assigned to the ν_1_ and ν_2_ stretches of carbonate [60], indicating the existence of minute impurities on the surface of nanoparticles generated by the acetate precursor [55]. Finally, the peak at 873 cm^−1^ could also be assigned to the Zn^2+^ presence in tetrahedral coordination [61,62].

The loading process of ZnO NPs with essential oils was monitored with the help of FTIR spectroscopy (Figure S1b). Specific peaks for the essential oils components were identified in Table 3.

Citronellal (32.2%), geraniol (19.6%), geranylacetate (15.1%), citronellol (6.4%), and citronellylacetate (5.5%) were predominant in citronella essential oil, while the main ingredients of thyme essential oil were thymol (47.9%), γ-terpinene (31.3%) and p-cymene (8.5%). D-limonene was dominant in orange (94.9%), grapefruit (93.2%), and limette (42.5%) essential oils, with linalool (3%), β-myrcene (2.5%), or γ-terpinene (15.4%) and α/β-pinene (15.7%) as minor components in these oils. Limonene (59%) was also the major component of bergamot essential oil, along linalyl acetate (17.1%), linalool (9.5%), and β-pinene (5%).

Lavender essential oil contained an important amount of linalool (44.2%), linalyl acetate (30%), and terpinen-4-ol (11%). Whilst, the main component of cinnamon essential oil was (E)-cinnamaldehyde (72%) mixed with smaller quantities of linalool (7%), β-caryophyllene (6.5%), eucalyptol (5.5%), and eugenol (4.5%). A more equilibrated composition was found in rosemary essential oil—containing borneol (23.7%), α-pinene (9.9%), α-caryophyllene (8.1%), ledol (6.5%), eucalyptol (4.9%), camphor (4.9%), and γ-terpinene (4.5%)—and minzol essential oil that comprised menthol (45.4%), menthone (16.1%), menthofuran (8.9%), cis-carane (8.7%), 1,8-cineole (4.5%), and neo-menthol (4.2%).

The FTIR spectra showed mostly the typical signals of the terpene and associated functional groups.

**Figure 5:**
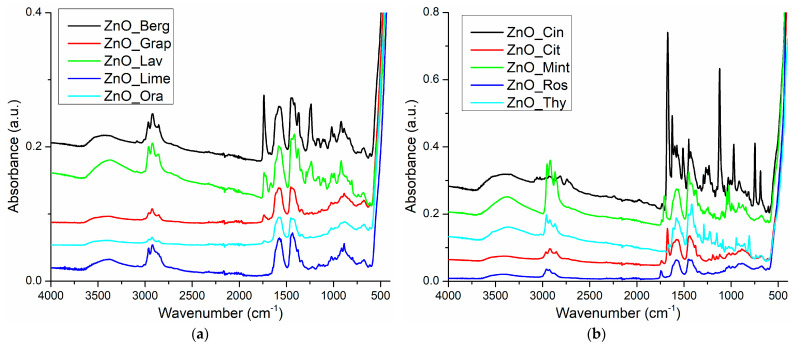
The FTIR spectra for the ZnO NPs loaded with essential oils (**a**) and (**b**).

The peaks from 3340 to 3430 cm^−1^ were assigned to the –OH stretching vibration from the alcohol, phenol, and carboxylic moieties. The peaks around 3000 cm^−1^ were from C–H stretching vibrations. Those from 3000 to 3030 cm^−1^ indicated the presence of unsaturated carbons Csp2–H like in thymol or γ-terpinene. Peaks in the region of 2800–3000 cm^−1^ were assigned to the methyl and methylene C–H symmetric and asymmetric vibrations and originated from all organic compounds (Figure 5a,b).

A distinct peak also was observed at ~1706/1743 cm^−1^, which could be attributed to the acetate or aldo (C=O) stretching vibration. These groups were present in geranylacetate/ citronellylacetate and citronellal, active ingredients of the citronella oil.

The peaks from 1620 to 1680 cm^−1^ were originating from C=C bonds, isolated or conjugated like in linalool or cinnamaldehyde from cinnamon essential oil. The signal of C=C–C stretching was responsible for the maximum from 1570 to 1585 cm^−1^ [63,64] (Figure S1c,d, Table 4).

C–H symmetrical and asymmetrical bending was the cause for the strong signal in the range of 1417–1450 cm^−1^.

Finally, the –C–O– stretching and –C–OH deformation vibration of alcohols generated the signals from 1021–1121 cm^−1^, including menthol in minzol essential oil or linalool and eugenol from cinnamon essential oil [65].

Due to the loading process, interactions appeared between essential oil components and the surface of nanoparticles, on which Zn^2+^ represented positive centers with electron deficit and O^2−^ as high electron density centers. Therefore, small modifications in the position of Zn–O and Zn–OH bands were noticeable in FTIR spectra of the essential-oils-loaded samples when compared with simple ZnO NPs.

### 3.4. Thermal Analysis (TG-DSC)

The thermal analysis (Figure S2) confirmed the synthesis of ZnO NPs using the solvothermal method. The small mass loss recorded up to 900 °C (1.54%) was caused by the elimination of solvent molecules adsorbed on the surface and some acetate impurities [10]. Evaluation of the quantity of essential oil loaded on ZnO NPs, as obtained from the synthesis, was carried out with thermal analysis (TG-DSC), Figure S3. By implementing the FTIR evaluation of the evolved gases from TG-DSC, monitoring of the desorption process of active components can be achieved.

**Figure 6:**
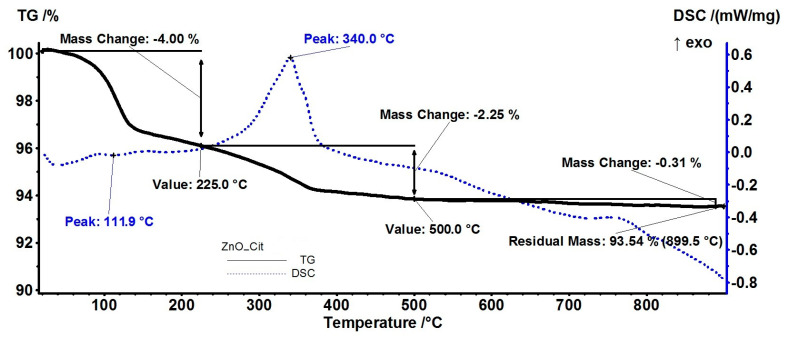

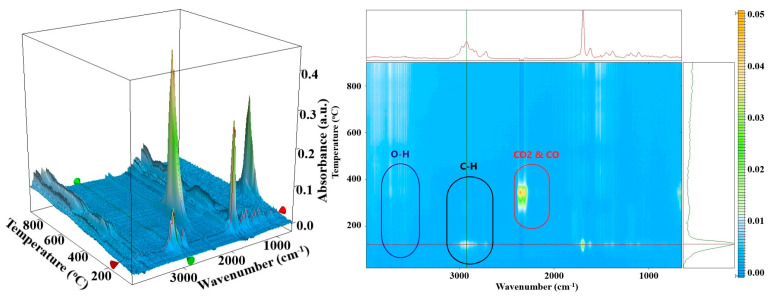
The TG-DSC curves for the ZnO_Cit sample, FTIR 3D diagram of the evolved gases, and its 2D projection in the wavenumber/temperature plan.

The thermal analysis of the ZnO_Cit sample presented a mass loss of 4.00% up to 225 °C accompanied by an endothermic effect with a minimum of 111.9 °C, indicating a desorption process (Figure 6). The upper part of the FTIR 2D projection presents the FTIR spectrum of the evolved gases at 115 °C, which could be assigned to the citronellal component [66]. The other less volatile components—geraniol, geranylacetate, and elemol—were eliminated in the second temperature interval of 225–500 °C, by oxidation and degradation, with a recorded mass loss of 2.25% [67]. An exothermic effect was associated with this process, with a maximum peak at 340.0 °C, indicating the presence of oxidative reactions. The FTIR 2D and 3D diagrams of evolved gases indicated the presence of carbon dioxide and water vapors in this temperature interval. On the right side of the FTIR 2D projection, there is a trace for hydrocarbon fragments (wavenumber 2925 cm^−1^) which presents a small peak at 350 °C indicating that fragmentation reaction also took place in this temperature interval. The estimated load of citronella essential oil was ~5%.

**Figure 7:**
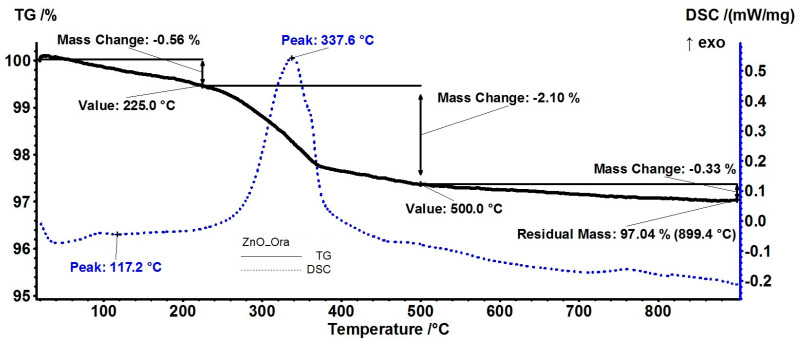

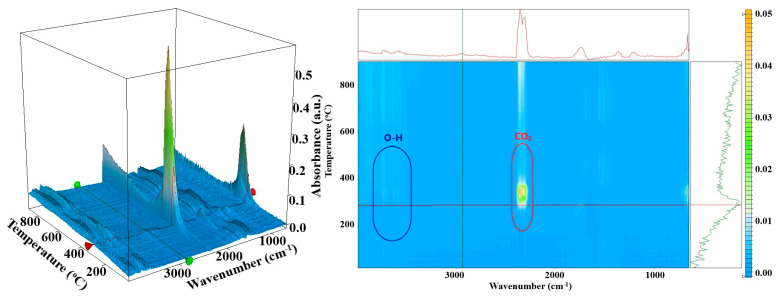
The TG-DSC curves, for the ZnO_Ora sample, FTIR 3D diagram of the evolved gases, and its 2D projection in the wavenumber/temperature plan.

**Figure 8:**
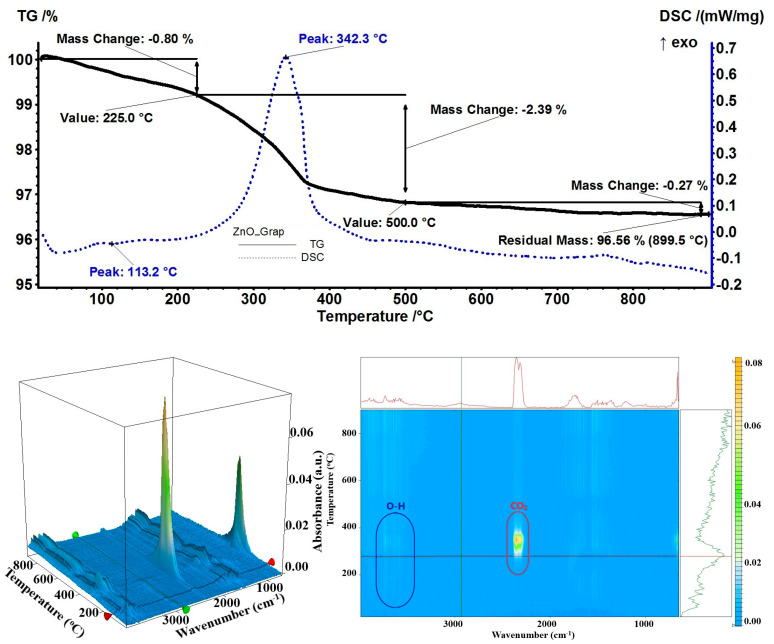
The TG-DSC curves, for the ZnO_Grap sample, FTIR 3D diagram of the evolved gases, and its 2D projection in the wavenumber/temperature plan.

The samples obtained from orange and grapefruit essential oils exhibited the lowest total mass loss due to the low persistence of the loaded essential oils (estimated load of 1.44% and 1.93%, respectively). As the major component of these two essential oils was limonene (over 90%), it likely did not create strong enough bonds with ZnO NPs to avoid its rapid release by evaporation [68]. Nevertheless, the other minor components of essential oils, like linalool or β-myrcene were still present in the sample [69,70], and their degradation was followed by the elimination of water vapors, carbon dioxide, and some traces of hydrocarbon fragments at over 300 °C, as presented in Figure 7 and Figure 8.

**Figure 9:**
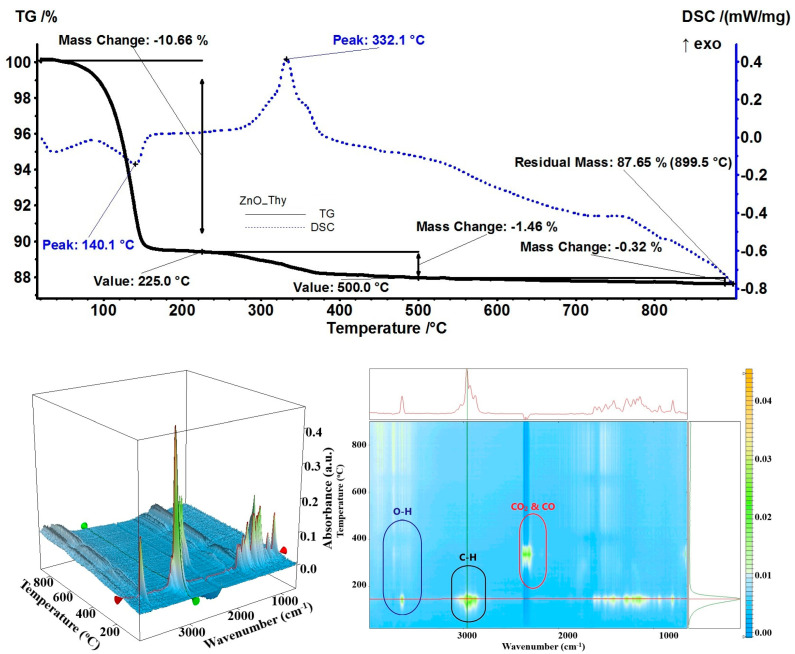
The TG-DSC curves, for the ZnO_Thy sample, FTIR 3D diagram of the evolved gases, and its 2D projection in the wavenumber/temperature plan.

The sample ZnO_Thy exhibited a mass loss of 10.66% between RT-225 °C; the process being correlated with an endothermic effect on the DSC curve at 140.1 °C (Figure 9). The FTIR 2D and 3D diagrams of evolved gases indicated the presence of organic compounds in this temperature interval. On top of the 2D projection, the FTIR spectrum at 150 °C indicated mainly the presence of thymol in the evolved gases (both γ-terpinene and p-cymene have FTIR spectra that are masked by the one of thymol) [63,71]. Between 225 and 500 °C, the residual organics from the ZnO NPs were burned away—the recorded mass loss was 1.46% with an associated exothermic effect at 332.1 °C—and the only products identified in the evolved gases spectra were carbon dioxide and water vapors. The estimated load of thyme essential oil was 10.98%.

**Figure 10:**
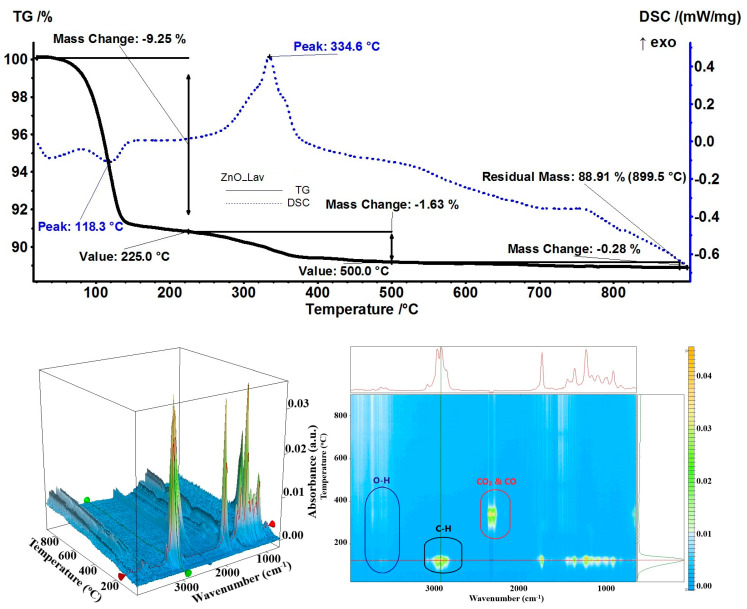
The TG-DSC curves, for the ZnO_Lav sample, FTIR 3D diagram of the evolved gases, and its 2D projection in the wavenumber/temperature plan.

Another sample with a high load of essential oil (9.70%) was ZnO_Lav. At a temperature up to 225 °C, the sample presented a mass loss of 9.25% with an endothermic effect at 118.3 °C. On top of the 2D projection from Figure 10, the FTIR of evolved gases at 125 °C showed a mixture of linalool and linalyl acetate, as indicated by the presence of –OH, C–H, and C=O vibrations at 3566, ~3000, and 1753 cm^−1^, respectively [72]. The sample lost an additional 1.63% in the temperature interval of 225–500 °C by oxidation of residual organics, as indicated by the presence of specific peaks of carbon dioxide and water vapors from the FTIR diagram of the evolved gases around 350 °C. The residual mass at 900 °C represented 88.91% which permitted evaluation of loaded lavender essential oil at 9.25%.

**Figure 11:**
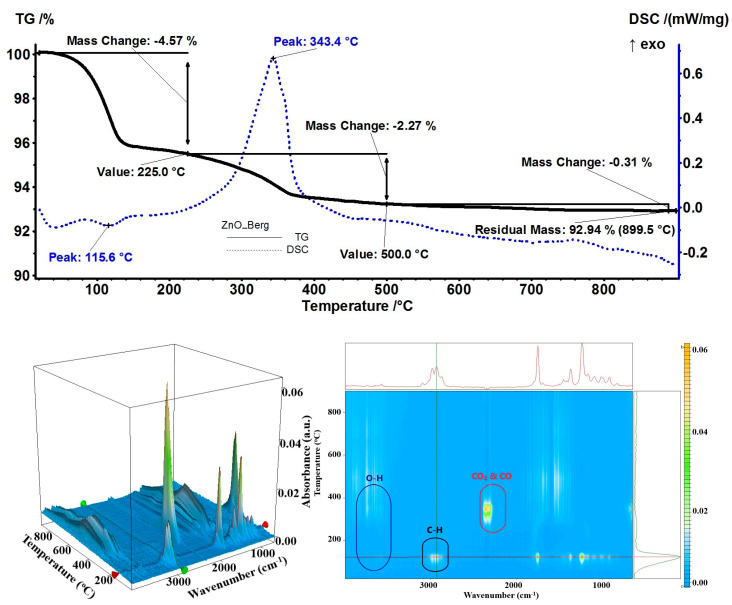
The TG-DSC curves, for the ZnO_Berg sample, FTIR 3D diagram of the evolved gases, and its 2D projection in the wavenumber/temperature plan.

Although the bergamot essential oil’s major component is limonene, and it has a low affinity for ZnO NPs as shown by samples with orange and grapefruit essential oils, the lower percentage (59%) in the essential oil composition indicates that the other components (linalyl acetate, linalool, or β-pinene) can still be observed [73]. Therefore, a 4.57% mass loss was recorded at the temperature up to 225 °C, as an endothermic process with a minimum at 115.6 °C on the DSC curve (Figure 11). The FTIR of evolved gases indicated the presence of linalyl acetate as C–H and C=O specific vibrations were identified (around 2864–3088 cm^−1^ and 1753 cm^−1^, respectively). In the interval of 225–500 °C, the rest of the organic compounds were burned away from the NP surface as indicated by the FTIR of the evolved gases (carbon monoxide, carbon dioxide, and water-specific vibrations were identified at 2177, 2355, 3566 cm^−1^, respectively). The residual mass of 92.94% was used to evaluate the loading of bergamot essential oil at 5.61%.

**Figure 12:**
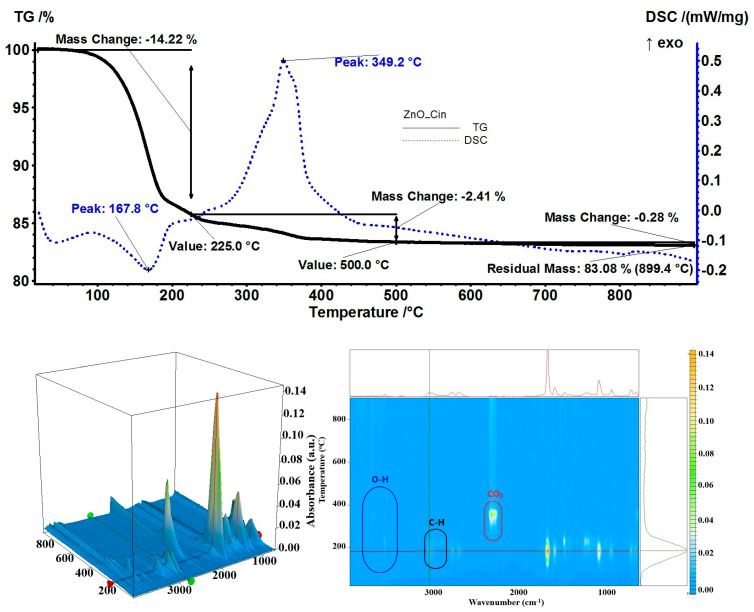
The TG-DSC curves, for the ZnO_Cin sample, FTIR 3D diagram of the evolved gases, and its 2D projection in the wavenumber/temperature plan.

The highest loading capacity, at 15.62%, calculated from the residual mass of 83.08%, was obtained from the sample with cinnamon essential oil. In the temperature interval of 20–225 °C, the sample exhibited a mass loss of 14.22% accompanied by an endothermic effect at 167.8 °C (Figure 12). The FTIR of evolved gases assessed the presence of O–H, C–H, and C=O bonds, which were consistent with cinnamaldehyde from the essential oil, with a maximum at 183 °C, while the maximum for eugenol was observed at 230 °C [74]. At the temperature of up to 500 °C, the sample presented a mass loss of 2.41% due to the oxidation of remaining organic compounds as indicated by the strong exothermic effect from 349.2 °C; at this temperature, the evolved gases were composed of carbon dioxide and water vapors, confirming the complete burning process.

**Figure 13:**
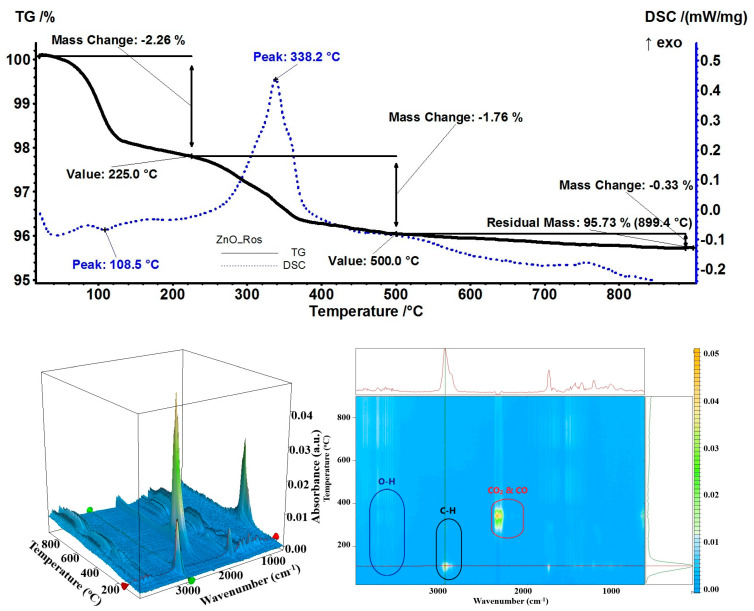
The TG-DSC curves, for the ZnO_Ros sample, FTIR 3D diagram of the evolved gases, and its 2D projection in the wavenumber/temperature plan.

For the sample with rosemary essential oil, a mass loss of 2.26% was recorded up to 225 °C. The desorption process was endothermic as indicated by the minimum of 108.5 °C on the DSC curve (Figure 13). The FTIR for evolved gases indicated the presence of borneol at 107 °C, quickly followed by α-caryophyllene at 112 °C [75]. The complete oxidation of remaining organic molecules occurred between 225 and 500 °C when a mass loss of 1.76% was recorded, accompanied by a strong exothermic effect at 338.2 °C. The residual mass of 95.73% indicated that only a fraction of rosemary essential oil had been loaded on ZnO NPs, estimated at 2.77%.

**Figure 14:**
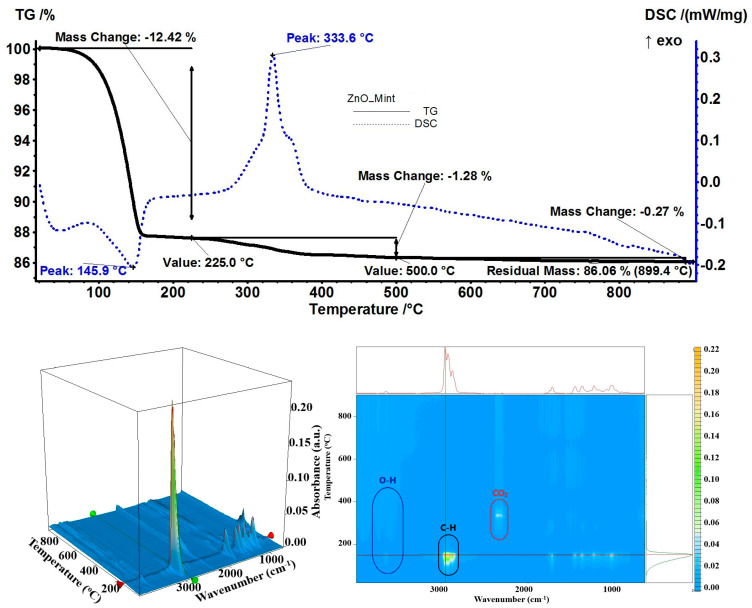
The TG-DSC curves, for the ZnO_Mint sample, FTIR 3D diagram of the evolved gases and its 2D projection in the wavenumber/temperature plan.

The sample ZnO_Mint exhibited the second-highest estimated load of 12.60%—most probably due to menthol’s high affinity for ZnO. The mass loss at the temperature up to 225 °C was 12.42%, with the desorption endothermic effect presenting the minimum at 145.9 °C (Figure 14). Both menthone and menthol were eliminated rapidly one after the other—menthone first—and the evolved gases FTIR indicated maximum peaks at 140 °C and 150 °C [76]. The elimination of residual organics took place after 225 °C when a mass loss of 1.28% was recorded together with a sharp and strong exothermic effect at 333.6 °C on the DSC curve. For this sample, the residual mass was 86.06%.

**Figure 15:**
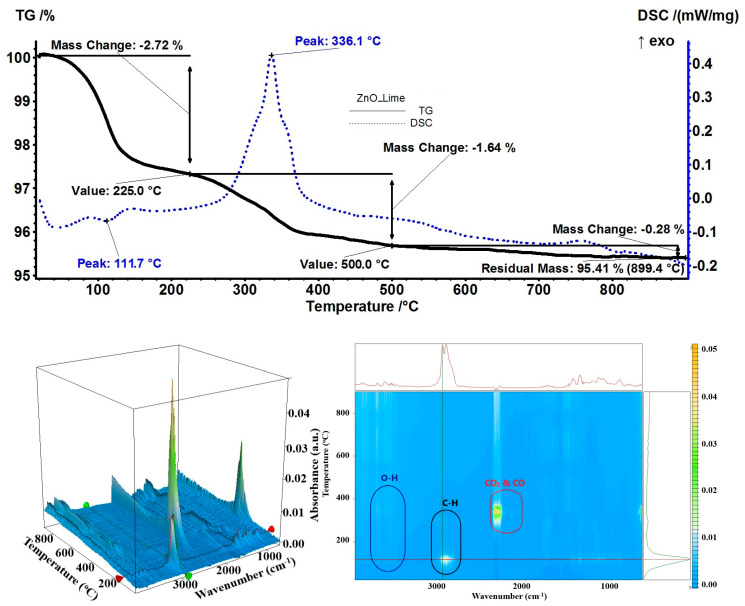
The TG-DSC curves, for the ZnO_Lime sample, FTIR 3D diagram of the evolved gases, and its 2D projection in the wavenumber/temperature plan.

As limette essential oil contains limonene and has a low affinity for the NPs, the estimated load was only 3.10%, based on the residual mass of 95.41%. The recorded mass loss at the temperature up to 225 °C was 2.72%, with an additional 1.64% up to 500 °C (Figure 15). In the first step, volatile organics were desorbed from the ZnO NP surfaces, as indicated by the endothermic effect from 111.7 °C, while in the second step, residual organic compounds were oxidized as indicated by the exothermic effect from 336.1 °C. The FTIR of evolved gases indicated the presence of γ-terpinene and β-pinene (C–H stretching vibrations around 3000 cm^−1^, for both sp^3^ and sp^2^ carbons; C=C stretching vibration at 1652 cm^−1^; C–H bending vibration at 1442 cm^−1^; and C=C bending vibration at 912 cm^−1^) [77,78]. The temperature at which these compounds were identified with the FTIR of evolved gases was 116 °C.

The principal numeric data from thermal analysis are presented in Table 5.

To conclude, the limonene had a low affinity for ZnO NPs and a high volatility leading to a low loading of samples with essential oils containing limonene, such as orange or grapefruit—both with under 2% load. The limonene represents around half of the mass of the bergamot or limette essential oil; therefore, the overall loading was higher due to the remaining compounds like linalyl acetate and linalool or γ-terpinene and α/β-pinene, respectively. The rosemary oil also exhibited a low loading capacity of only 2.77% due to the high content of non-polar hydrocarbons and low content of bulky alcohols.

For the citronella essential oil, a medium loading capacity of 5% was found as compounds like citronellal, geraniol or geranylacetate are more polar due to functional oxygen-based moieties, and therefore exhibited a higher affinity towards ZnO NPs.

The high-affinity phenols and alcohols for ZnO NPs led to high loading of essential oils that contain such compounds as major components: thymol in thyme oil, linalool in lavender oil, and menthol in minzol oil. The presence of additional menthone in minzol oil led to a higher load of 12.60%. However, the highest load, 15.62%, was obtained from cinnamon oil due to the presence of (E)-cinnamaldehyde in a high quantity (72%) with the additional presence of linalool and eugenol.

The essential oil used to load ZnO NPs represents ~20% of each sample mass. However, none of the essential oils reached the theoretical value—the maximum load achieved being 15.62%. These results confirmed that some of the essential oil components (especially the non-polar ones) did not interact strongly enough with the ZnO NP surface to be retained.

### 3.5. Antibacterial Activity Assay

The disk-in-agar diffusion method was employed to evaluate the antibacterial activity of the essential oils-loaded ZnO NP samples. This diffusion-based method is considered suitable for identifying the most potent antibacterial agents. The zone of inhibition determined by the diffusion method is proportional to the bacterial susceptibility to the essential oil present in the paper disc. The zone of inhibition around the impregnated disc defines the extent of antimicrobial activity and is determined as zone diameter in mm (which includes the size of the paper disc). The factors that affect the diameter of this zone are bacterial cell growth and the rate of diffusion.

The results employing the diffusion method are shown in Table 6. The evaluation consisted of assessing the size of the zones of inhibition induced by ZnO NPs loaded with essential oils, and areas where microbial growth was absent. The tests were performed in triplicates and the results were reported as the average diameter. These are directly proportional to the sensitivity of the germ used; the more active the substance in the structure of the tested sample, the wider the zone of inhibition of microbial development. The results were read after 24 h of incubation at 37 °C.

ZnO NPs showed a moderate inhibitory effect on the tested bacteria, but the inhibitory activity was more pronounced in combination with the essential oils, thus demonstrating their synergistic effect. The synergistic activity between ZnO NPs and the studied essential oils was most visible, especially for citronella, thyme, and cinnamon essential oils against all the bacterial strains. The antibacterial activity of ZnO was very evident in the case of *S. typhimurium*, followed by *E. coli* and *L. monocytogenes*, demonstrating that ZnO has significant antibacterial potential.

All combinations of ZnO with essential oils showed antimicrobial activity on the test microorganism strains by direct contact with the environment. Samples proved less active against Gram-positive bacteria than Gram-negative ones (*S.aureus* being the most resistant strain), but the differences in sensitivity were not distinct.

**Figure 16:**
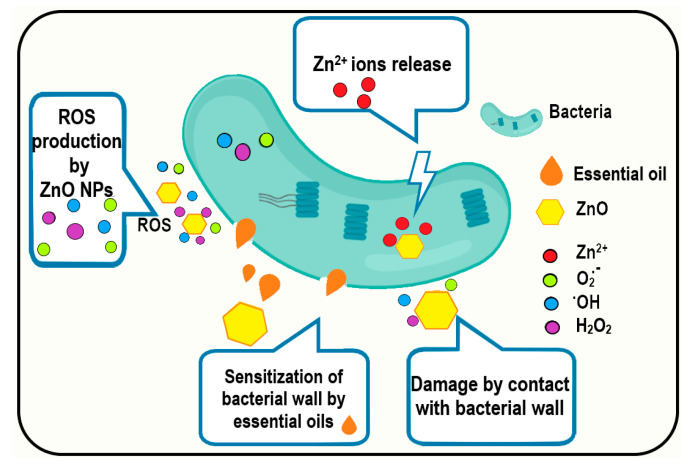
The proposed mechanism for the antibacterial activity of ZnO-EO NPs.

The inhibitory effect of ZnO on *S. aureus* was relatively weak (8.7 mm of inhibition diameter), but seven samples loaded with essential oils presented a significant increase in the inhibition zone. For *S. aureus* strain, only the ZnO NPs loaded with orange, grapefruit, and limette essential oils presented a negligible increase in the diameter of the inhibition zone—these being also the samples with the smallest EO load. For all the other samples, the presence of essential oils along ZnO NPs led to increases in inhibition zones, including samples containing bergamot and rosemary essential oils, which exhibited only 7–9 mm diameter of inhibition zones as stand-alone substances; this was likely because the essential oils were sensitizing the bacterial cell to become less resistant to ZnO NPs (Figure 16). The mechanism could be related to the presence of essential oils, which changed the concentration of adenosine triphosphate and/or hyperpolarization of the cell wall, leading to a decrease in the cytoplasmic pH [79], which in turn made it easier for the ZnO NPs to damage the membrane, and zinc ions to bind the proteins and enzymes, disrupting vital processes inside the cell [80]. The combined synergistic antibacterial activity of ZnO and essential oils was demonstrated in this study against both Gram-positive and Gram-negative bacteria.

Overall, the ZnO NP samples loaded with thyme, citronella, and cinnamon oils were the most effective, showing significant areas of inhibition of bacterial growth. We have previously reported strong antimicrobial activity of chitosan or alginate-based films with ZnO and citronella essential oil [13,67]. The literature also reported some antibacterial nanocomposites with thyme essential oil used in specific applications [81,82]. In addition, some packaging films containing ZnO and cinnamon essential oils have been reported lately [83,84]. The antimicrobial activity of ZnO NPs loaded with bergamot [85], orange [86], grapefruit, limette, or minzol essential oils is not well studied. Nevertheless, some studies directly used limonene instead of oils to obtain antimicrobial packaging [87]. Similarly, there are some reports on ZnO loaded directly with menthol for wound dressing applications [88].

The loaded nanoparticles can be further used with a polymer matrix to obtain innovative composite materials with specific applications like wound dressing or food packaging [33,89,90,91], tailoring them for prolonged release of the loaded essential oils or for rapid, burst desorption.

## 4. Conclusions

The obtained ZnO NPs presented good antibacterial activity on all four bacterial strains (better on Gram-negative than on Gram-positive bacteria), supporting further use in medical applications.

In this study, we loaded ZnO NPs with ten different essential oils, and the antibacterial activity of the samples was determined. Due to composition and high volatility, some essential oils exhibited low loading capacity, especially those containing non-substituted terpenes like limonene (e.g., 1.44% estimated load for orange essential oil). At the same time, the essential oils containing alcohol, aldehyde, and ketone derivatives presented a higher loading capacity (e.g., 15.62% for cinnamon essential oil). Synergic antibacterial activity between ZnO NPs and essential oils was observed for most of the samples, related to the effective load on the surface of ZnO NPs. Therefore, samples with a higher content of strong antibacterial oils like thyme, cinnamon, or citronella essential oils present a higher potential for antibacterial applications (diameter of inhibition zone higher than 15 mm) like wound dressing or food packaging.

## Figures and Tables

**Table 1:** Sample code label for each essential oil.

Sample Label	Essential Oil Used
ZnO	-
ZnO_Cit	Citronella
ZnO_Ora	Orange
ZnO_Thy	Thyme
ZnO_Lav	Lavender
ZnO_Grap	Grapefruit
ZnO_Berg	Bergamot
ZnO_Cin	Cinnamon
ZnO_Ros	Rosemary
ZnO_Mint	Minzol
ZnO_Lime	Limette

**Table 2:** Bacteria strains used in this study.

No	Bacteria Strains	Gram-Positive	Gram-Negative
1	*Listeria monocytogenes,* ATCC 19114	Yes	
2	*Staphylococcus aureus*, ATCC 25923	Yes	
3	*Salmonella typhimurium*, ATCC 14028		Yes
4	*Escherichia coli*, ATCC 25922		Yes

**Table 3:** Lattice parameters for obtained ZnO nanopowder.

Unit Cell	*a* = *b* [Å]	*c* [Å]	V [Å^3^]	*c/a*	Microstrain (%)	Average Crystallite Size (nm)	Dislocation Density (δ) ×10^−4^
ZnO	3.25081	5.20661	47.65065	1.60163	0.26 ± 0.10	34.45 ± 4.93	8.43

**Table 4:** Principal FTIR peaks of ZnO-essential oil samples and their assignment.

Sample/ Wavenumber (cm^−1^)	ZnO	ZnO_ Cit	ZnO_ Ora	ZnO_ Thy	ZnO_ Lav	ZnO_ Grap	ZnO_ Berg	ZnO_ Cin	ZnO_ Ros	ZnO_ Mint	ZnO_ Lime
–OH	3401	3340	3414	3374	3392	3423	3406	3379	3423	3379	3388
–C_sp2_–H	-	-	-	3020	3006	3010	3011	3027	-	-	3009
–C_sp3_–H CH_3 asym_ CH_2 asym_ CH_3 sym_ CH_2 sym_	- - - - -	2965 2920 2854 2833	2959 2926 2868 2854	2958 2923 2868 -tail	2962 2925 2868 2855	2961 2927 2871 2855	2965 2921 2872 2856	2975 2930 2842 2813	2959 2927 2873 2854	2952 2918 2868 2847	2962 2923 2855 2832
C=O	-	1735	1740	1706	1735	1735	1735	1733	1743	1706/38	-
C=C–C _stretching_	-	1674	1620	1620	1622/74	1620	1622	1622/73	1620	1620	1620
	-	1572	1584	1584	1575	1572	1572	1572	1584	1584	1584
C–O–_str_ C–OH_def_	-	1121	-	-	1117	1121	1115	1121	1045	1045	1045
		1020	1024	1024	1020	1020	1020	1025	1024	1024	1024
−_bending_	615/673	612/677	614/680	612/677	613/682	613/680	612/678	606/688	613/677	612/678	614/678
Zn–O_str_	458	456	420	418	424	420	422	455	456	455	417

**Table 5:** Temperature intervals for the principal mass loss processes, and associated thermal effects.

Sample	Mass Loss (%) RT-225 °C	Endothermic Peak (°C)	Mass Loss (%) 225–500 °C	Exothermic Peak (°C)	Residual Mass (%)	Estimated Load (%)
ZnO	0.11%	70.1	1.03%	328.2	98.46%	-
ZnO_Cit	4.00%	111.9	2.25%	340.0	93.54%	5.00%
ZnO_Ora	0.56%	117.2	2.10%	337.6	97.04%	1.44%
ZnO_Thy	10.66%	140.1	1.46%	332.1	87.65%	10.98%
ZnO_Lav	9.25%	118.3	1.63%	334.6	88.91%	9.70%
ZnO_Grap	0.80%	113.2	2.39%	342.3	96.56%	1.93%
ZnO_Berg	4.57%	115.6	2.27%	343.4	92.94%	5.61%
ZnO_Cin	14.22%	167.8	2.41%	349.2	83.08%	15.62%
ZnO_Ros	2.26%	108.5	1.76%	338.2	95.73%	2.77%
ZnO_Mint	12.42%	145.9	1.28%	333.6	86.06%	12.60%
ZnO_Lime	2.72%	111.7	1.64	336.1	95.41%	3.10%

**Table 6:** Diameters of zones of inhibition (mm *).

Sample	Gram-Positive Bacteria		Gram-Negative Bacteria	
	*L. monocytogenes*	*S. aureus*	*S. typhimurium*	*E. coli*
ZnO_Cit	12.3 ± 0.6 ^b^	12.0 ± 1.0 ^b^	16.3 ± 0.6 ^b^	14.7 ± 0.6 ^d^
ZnO_Ora	11.0 ± 0.0 ^a^	8.7 ± 0.6 ^a^	15.0 ± 0.0 ^a^	10.7 ± 0.6 ^a^
ZnO_Thy	13.7 ± 0.6 ^c^	14.3 ± 0.6 ^c^	18.3 ± 0.6 ^c^	15.7 ± 0.6 ^d^
ZnO_Lav	10.7 ± 0.0 ^a^	12.7 ± 0.6 ^b^	14.7 ± 0.0 ^a^	11.7 ± 0.6 ^b^
ZnO_Grap	10.7 ± 0.6 ^a^	9.0 ± 0.0 ^a^	14.3 ± 0.0 ^a^	10.3 ± 0.6 ^a^
ZnO_Berg	13.3 ± 0.6 ^b,c^	12.7 ± 0.6 ^b^	13.0 ± 1.0 ^d^	12.0 ± 0.0 ^b^
ZnO_Cin	13.7 ± 0.6 ^c^	12.0 ± 0.6 ^b^	16.0 ± 0.0 ^b^	13.3 ± 0.6 ^c^
ZnO_Ros	11.0 ± 0.0 ^a^	12.3 ± 0.6 ^b^	11.7 ± 0.6 ^d^	13.0 ± 1.0 ^b,c^
ZnO_Mint	11.3 ± 0.6 ^a^	12.0 ± 0.0 ^b^	12.0 ± 1.0 ^d^	11.7 ± 0.6 ^b^
ZnO_Lime	10.3 ± 0.6 ^a^	9.3 ± 0.6 ^a^	12.3 ± 0.6 ^d^	10.3 ± 0.6 ^a^
ZnO	10.7 ± 0.6 ^a^	8.7 ± 0.6 ^a^	14.7 ± 0.6 ^a^	10.3 ± 0.6 ^a^
Citronella	22.0 ± 1.0	16.3 ± 0.6	9.7 ± 0.6	37.7 ± 1.2
Orange	13.0 ± 1.0	9.7 ± 0.6	10.0 ± 0.0	9.7 ± 0.6
Thyme	24.3 ± 0.6	57.7 ± 0.6	55.3 ± 0.6	59.0 ± 1.0
Lavender	12.0 ± 0.0	20.3 ± 0.6	15.0 ± 1.0	17.0 ± 1.0
Grapefruit	12.3 ± 0.6	12.7 ± 0.6	11.7 ± 0.6	12.3 ± 0.6
Bergamot	8.0 ± 0.0	7.3 ± 0.6	8.7 ± 0.6	6.7 ± 0.6
Cinnamon	30.7 ± 0.6	31.7 ± 0.6	22.0 ± 1.0	15.3 ± 0.6
Rosemary	9.0 ± 0.0	9.0 ± 0.0	8.3 ± 0.6	11.7 ± 0.6
Minzol	15.0 ± 1.0	15.0 ± 1.0	13.0 ± 1.0	19.3 ± 0.6
Limette	7.3 ± 0.6	11.0 ± 1.0	9.0 ± 0.0	10.0 ± 1.0

* The diameter of the paper disc is included; mean value of three replicates ± standard deviation; ^a, b, c, d^ different small letters indicate statistically significant differences between samples on same column (*p* < 0.05).

## Data Availability

Not applicable.

## Supplementary Materials

The following supporting information can be downloaded at: https://www.mdpi.com/article/10.3390/pharmaceutics15102470/s1, Figure S1: The FTIR spectra for the ZnO NPs (a) and ZnO NPs loaded with essential oils (b); detail of the 1000–1800 cm^−1^ zone (c) and (d); Figure S2. The TG-DSC curves for the ZnO NPs; Figure S3. The TG curves for the ZnO NPs and ZnO NPs loaded with essential oils
