# Supplementary material for: Antibacterial Activity of Zinc Oxide Nanoparticles Loaded with Essential Oils
Source: Pharmaceutics. 2023 Oct 15;15(10):2470. doi: 10.3390/pharmaceutics15102470 (PMC10610287; doi:10.3390/pharmaceutics15102470)
Supplement: Supplementary file 1 [file pharmaceutics-15-02470-s001.zip › pharmaceutics-2605963-supplementary.pdf]

pharmaceutics

MDPI

Article

Antibacterial Activity of Zinc Oxide Nanoparticles Loaded with Essential Oils

Ludmila Motelica [1,2], Bogdan-Stefan Vasile [1,2,3], Anton Ficai [1,2,3,4], Adrian-Vasile Surdu [1,2,3], Denisa Ficai [1,2,3], Ovidiu–Cristian Oprea [1,2,3,4]*, Ecaterina Andronescu [1,2,3,4], Gabriel Mustățea[5], Elena Loredana Ungureanu[5] and Alina Alexandra Dobre[5]

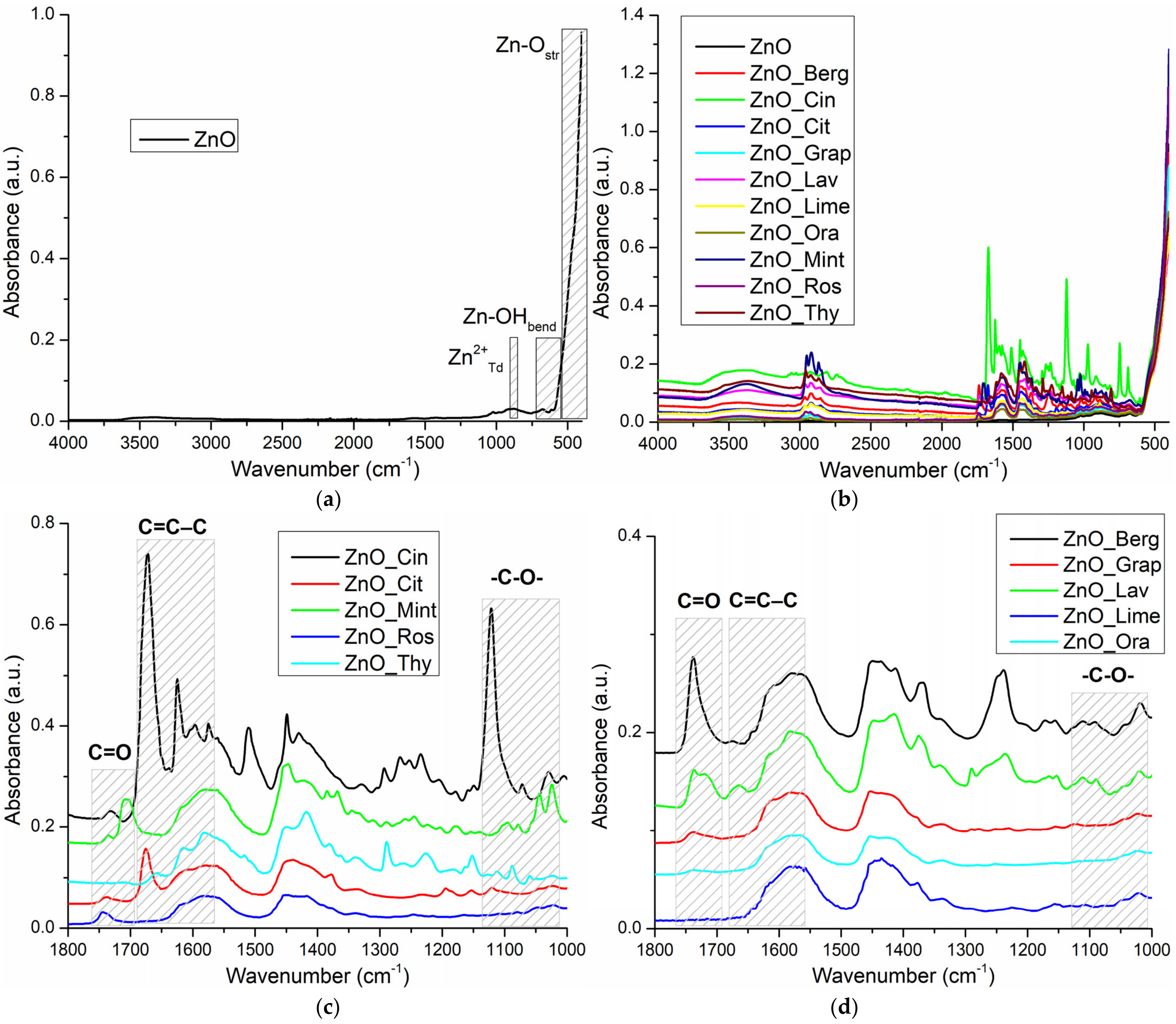

Figure S1. The FTIR spectra for the ZnO NPs (**a**) and ZnO NPs loaded with essential oils (**b**); detail of the 1000-1800 cm^{-1} zone (**c**) and (**d**).

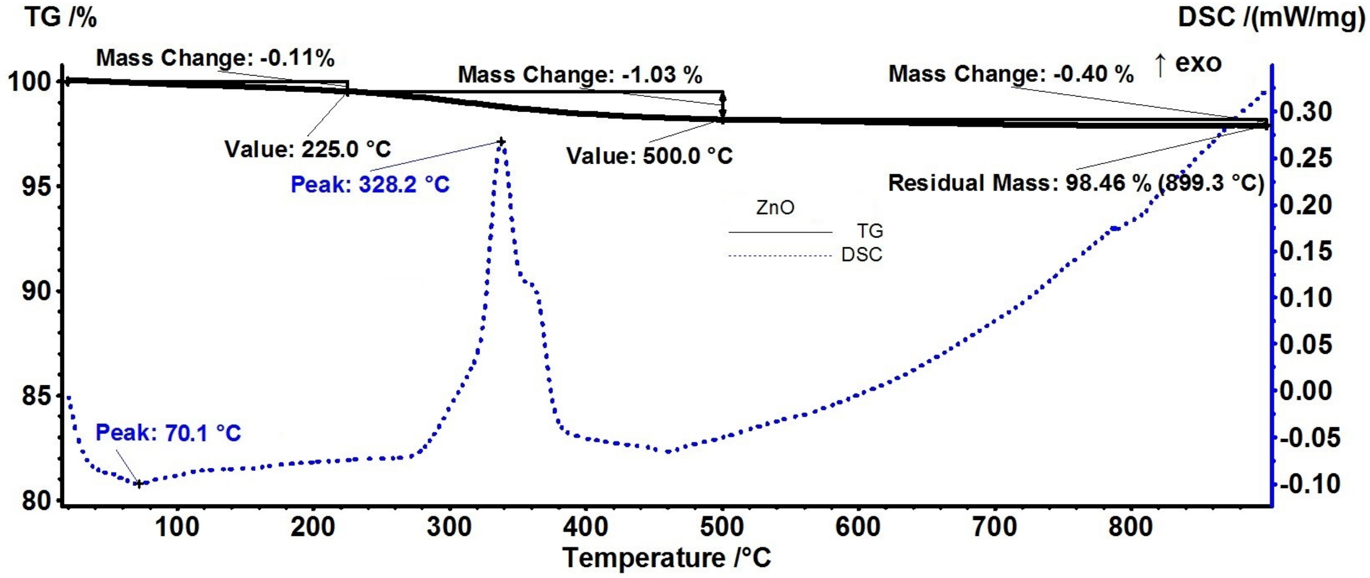

Figure S2. The TG-DSC curves for the ZnO NPs

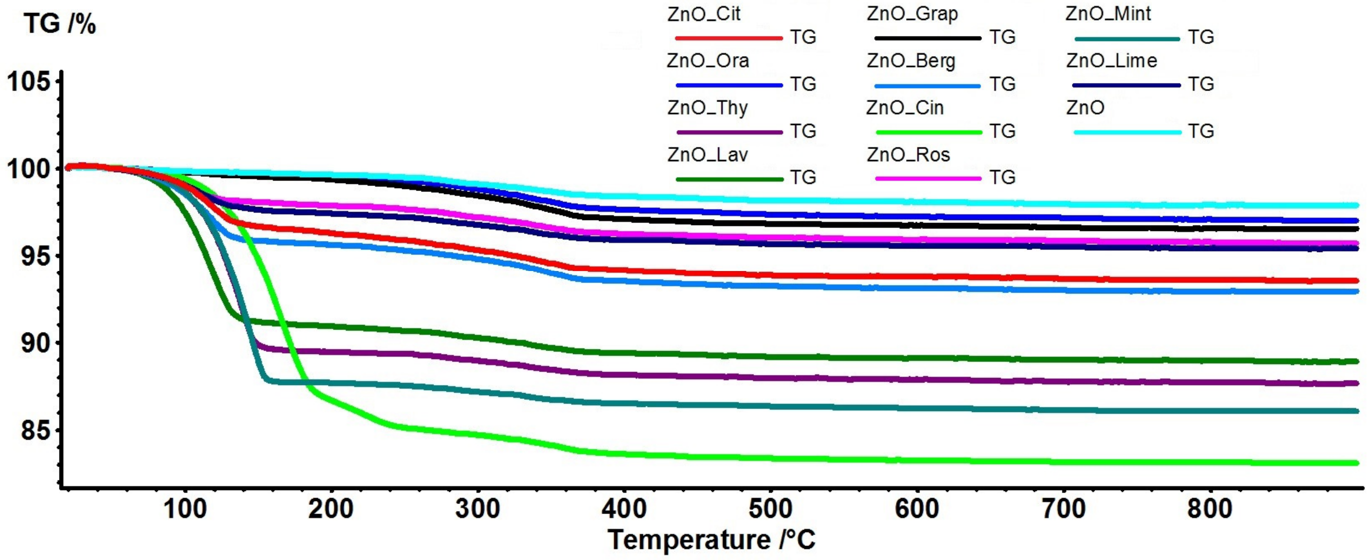

Figure S3. The TG curves for the ZnO NPs and ZnO NPs loaded with essential oils
